# Effects of hypoxia and hyperoxia on the differential expression of VEGF-A isoforms and receptors in Idiopathic Pulmonary Fibrosis (IPF)

**DOI:** 10.1186/s12931-017-0711-x

**Published:** 2018-01-15

**Authors:** Shaney L. Barratt, Thomas Blythe, Khadija Ourradi, Caroline Jarrett, Gavin I. Welsh, David O. Bates, Ann B. Millar

**Affiliations:** 1 Academic Respiratory Unit, Learning and Research Building, Southmead Hospital, Bristol, UK; 2 Bristol Renal, Translational Health Sciences, Bristol Medical School, University of Bristol, Bristol, UK; 3 Cancer Biology, Division of Cancer and Stem Cells, School of Medicine, University of Nottingham, Nottingham, UK

**Keywords:** Interstitial lung disease, Vascular endothelial growth factor, Hypoxia, Idiopathic pulmonary fibrosis

## Abstract

Dysregulation of VEGF-A bioavailability has been implicated in the development of lung injury/fibrosis, exemplified by Idiopathic Pulmonary Fibrosis (IPF). VEGF-A is a target of the hypoxic response via its translational regulation by HIF-1α. The role of hypoxia and hyperoxia in the development and progression of IPF has not been explored. In normal lung (NF) and IPF-derived fibroblasts (FF) VEGF-A_xxx_a protein expression was upregulated by hypoxia, mediated through activation of VEGF-A_xxx_a gene transcription. VEGF-A receptors and co-receptors were differentially expressed by hypoxia and hyperoxia. Our data supports a potential role for hypoxia, hyperoxia and VEGF-A_xxx_a isoforms as drivers of fibrogenesis.

**Electronic supplementary material:**

The online version of this article (10.1186/s12931-017-0711-x) contains supplementary material, which is available to authorized users.

## Introduction

Oxygen homeostasis is considered important for the maintenance of normal lung structure and function [1]. Vascular Endothelial Growth Factor–A (VEGF-A) is transcriptionally regulated by oxygen tension variations, via Hypoxia Inducible Factor-1 α (HIF-1α) [2]. The VEGF-A gene is differentially spliced to produce several functioning isoforms, the subscript number denoting the number of amino acids in the protein [3]. Proximal splicing in exon 8 produces the conventional ‘angiogenic’ family of isoforms (VEGF-A_xxx_a) whilst distal splice site selection produces a second ‘inhibitory’ family (VEGF-A_xxx_b) [4, 5]. Of these two families, VEGF-A_165_a and VEGF-A_165_b are the most widely studied isoforms. In the context of Idiopathic Pulmonary Fibrosis (IPF), we recently proposed that co-ordinated expression of the differentially spliced, VEGF-A_xxx_a and VEGF-A_xxx_b isoforms, is important in disease pathogenesis; alveolar epithelial type II (ATII) cell-derived VEGF-A_xxx_a acting as a driver of the fibrotic process, with protective or anti-fibrotic properties of VEGF-A_xxx_b [6].

Dense areas of lung fibrosis have been shown to be hypoxic [7] and high flow oxygen is used therapeutically in these conditions but the role of hypoxia and hyperoxia in the development and progression of IPF is unknown. We investigated the effect of hypoxia and hyperoxia on VEGF receptor, co-receptor and VEGF-A isoform expression in fibroblasts explanted from histologically normal lung (normal fibroblasts, NF) compared to those from IPF tissue (fibrotic fibroblasts, FF).

## Methods

Methodology and statistical analyses are available in Additional file 1. Additional file 2: Figure S1 details the primer sequences used to perform quantitative Reverse Transcriptase Polymerase Chain Reactions (qRTPCR).

## Results

Cobalt Chloride induced hypoxic-like growth conditions in NF and FF cultures, evidenced by HIF-1α expression, which was absent in normoxic conditions. Interestingly, hypoxia induced significantly less HIF-1α expression in FF than NF (See Additional file 3: Figure S2a).

We examined the effect of hypoxia and hyperoxia on fibroblast synthetic function. Hypoxia stimulated NF and FF fibronectin mRNA to the same extent, whilst hyperoxia significantly up-regulated fibronectin mRNA in FF only. In contrast, there was no significant effect of either hypoxia or hyperoxia on fibroblast pro-collagen-1α (See Additional file 3: Figure S2b).

**Fig. 1:**
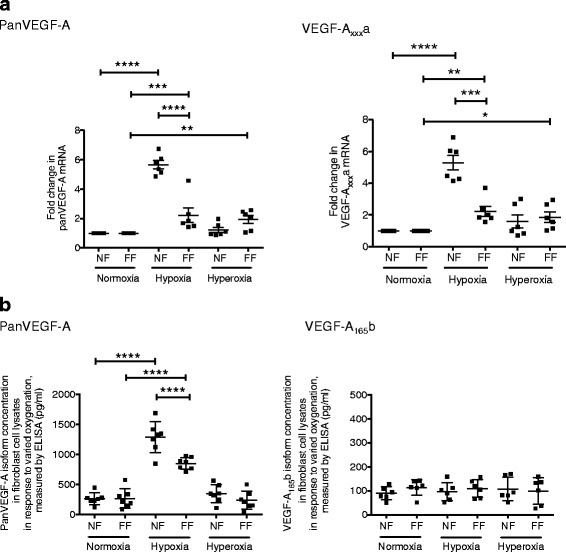
**a** Vascular endothelial growth factor-A (VEGF-A) mRNA levels in Normal (NF) and Fibrotic (FF) fibroblast total RNA lysates in response to 24 h exposure to hypoxia and hyperoxia. Using quantitative reverse transcriptase polymerase chain reactions (qRT-PCR) of total RNA cell lysates, panVEGF-A mRNA levels significantly increased in response to hypoxia in NF (****p < 0.0001) and FF (***p < 0.001). The response of FF to hypoxia was significantly attenuated compared to the NF response (****p < 0.0001). FF panVEGF-A mRNA levels also significantly increased in response to hyperoxia (*p* < 0.01), (NF and FF *n* = 6). Changes in VEGF-A_xxx_a mRNA levels reflected those seen for panVEGF-A with increased mRNA levels in response to hypoxia in total RNA lysates of NF (****p < 0.0001) and FF (*p* < 0.01), an attenuated response of FF to hypoxia compared to NF (***p < 0.001) and increased FF VEGF-A_xxx_a mRNA levels in response to hyperoxia (*p < 0.05), (NF and FF *n* = 6). In contrast, VEGF-A_xxx_b mRNA levels did not change in response to hypoxia or hyperoxia (NF and FF *n* = 6) (data not shown). Data are presented as mean fold change in expression (2^△△CT) with SEM, data analysis performed on △△CT values. Statistical analysis: analysis of variance with post hoc Holm-Sidak multiple comparisons analysis used throughout. **b** VEGF-A isoform protein expression in Normal (NF) and Fibrotic (FF) fibroblast cell lysates in response to 24 h exposure to hypoxia and hyperoxia. By ELISA, panVEGF-A protein expression in NF and FF cell lysates were significantly up-regulated in response to hypoxia (****p < 0.0001). The response of FF to hypoxia was significantly attenuated compared to the NF response (****p < 0.0001). Hyperoxia had no significant effect on panVEGF-A protein expression in NF and FF (NF *n* = 7, FF *n* = 7). Using a specific VEGF-A_165_b ELISA, there was no significant effect of either hypoxia and hyperoxia on VEGF-A_165_b expression in both NF and FF (NF and FF *n* = 6). Data presented as means with SEM, ANOVA with Holm’s Sidak multiple comparisons statistical analysis

We then explored the effect of hypoxia and hyperoxia on VEGF-A isoform expression. PanVEGF-A and VEGF-A_xxx_a mRNA levels were significantly up-regulated in response to hypoxia in NF and FF, although the FF response to hypoxia was significantly less than NF (Fig. 1a). Quantification of NF and FF panVEGF-A protein corroborated these findings (Fig. 1b). In contrast, hyperoxia up-regulated panVEGF-A and VEGF-A_xxx_a mRNA levels in FF but had no significant effect on NF or FF VEGF-A protein expression. Similarly, NF and FF VEGF-A_xxx_b mRNA (data not shown) and VEGF-A_165_b protein (Fig. 1b) were not significantly altered in response to hypoxia or hyperoxia. Collectively, these data support selective up-regulation of VEGF-A_xxx_a proteins in response to hypoxia, mediated in part through increased transcription.

Expression of VEGF-A receptors and co-receptors was then examined (See Additional file 4: Figure S3). VEGFR1 mRNA expression was significantly up-regulated in NF and FF in response to hypoxia, although variance in the FF response was evident (See Additional file 4: Figure S3a). In contrast, VEGFR1 protein expression was only significantly upregulated in response to hypoxia in NF, although a similar pattern was observed in FF in response to hypoxia (See Additional file 4: Figure S3b). Hyperoxia had no statistically significant effect on VEGFR1 expression in NF or FF.

VEGFR2 expression was absent in normoxic NF and FF by western blotting, with levels below the detection limit (30 pg/ml) of a specific VEGFR2 ELISA (data not shown), as previously described [6]. VEGFR2 expression was not stimulated by hypoxia or hyperoxia.

By contrast, hypoxia significantly down-regulated of NP1 mRNA and protein expression in NF but had no significant effect on FF. Hyperoxia up-regulated NP1 mRNA and protein expression in FF, but had no significant effect on NF (See Additional file 3: Figure S2c). Both NF and FF NP2 mRNA levels were up-regulated in response to hypoxia but changes did not translate to the protein level (See Additional file 4: Figure S3a and c). Finally, hyperoxia had no significant effect NF and FF NP2 co-receptor expression.

## Discussion

Conflicting roles for VEGF-A as both a contributory [6–8] and protective [9–11] factor in the development of IPF have been reported. We recently described a paradigm in which the co-ordinated expression of differentially spliced VEGF-A_xxx_a and VEGF-A_xxx_b isoforms is important for lung fibrogenesis [6], offering a potential explanation for these apparently conflicting reports. In this pre-clinical study of murine pulmonary fibrosis, specific deletion of all VEGF-A isoforms from ATII cells, over-expression of VEGF-A_165_b in ATII cells and intraperitoneal delivery of VEGF-A_165_b, all resulted in significant amelioration of the fibrotic response. Together these results indicated that it is the VEGF-A_xxx_a family that is profibrotic and the VEGF-A_xxx_b that is inhibitory/regulatory. Hypoxia and hyperoxia have been implicated in the development of IPF [12] and acute lung injury [13] respectively, but the hypoxia/hyperoxia-VEGF-A axis has not been examined previously in primary normal or IPF derived lung fibroblasts.

A hypoxic environment exists in IPF as demonstrated by HIF-1α expression in IPF but not in normal lung tissue [12]. Upregulation of VEGF-A in response to hypoxia has been reported in a variety of tissues and cell types [2]. In this study of NF and FF, hypoxia stimulated the expression of panVEGF-A but not VEGF-A_165_b proteins, inferring that specific upregulation of the VEGF-A_xxx_a isoforms occured and raises the possibility of preferential VEGF-A gene splicing towards VEGF-A_xxx_a isoform production during hypoxia. Since HIF1α levels were increased in the hypoxic culture conditions, and VEGF-A is known to be transcriptionally regulated by HIF-1α, the increased VEGF-A_xxx_a mRNA observed may have occurred in part due to HIF-1α activation of VEGF-A_xxx_a gene transcription. In IPF, the progressive nature of the condition results in new areas of tissue fibrosis developing, which theoretically could provide ongoing local production of HIF1α to drive this process.

We suggest that further work is required to explore the contribution of other known regulatory mechanisms of VEGF during hypoxia, such as the post-transcriptional stabilization of VEGF mRNA through the formation of a hypoxia-inducible protein complex at the 3’-UTR of the VEGF gene [14] and the relevance of the functional internal ribosome entry site which enables efficient cap-independent initiation of translation during hypoxia [15]. Furthermore, TGF-β is a potent pro-fibrotic cytokine that has been shown to co-operate synergistically with hypoxia in stimulating VEGF gene expression in several cell lines [16]. It would be interesting therefore to also investigate the differential production of VEGF-A splice isoforms in response to TGF-β stimulation.

The response to hypoxia was significantly attenuated in FF compared to NF, which may reflect a reduction in HIF-1α-mediated activation of VEGF-A gene transcription and implies that the FF were less responsive to hypoxia. In vitro*,* hypoxia stimulated fibronectin mRNA levels in NF and FF. In the context of our previous work [6] and others [12], it provides additional support for both hypoxia and VEGF-A_xxx_a isoforms as drivers of fibrogenesis. We suggest that the observed blunted response of FF may be due to exposure to chronic hypoxia in the context of IPF. This may allow the FF responses to be overwhelmed by those from more ‘normal areas’ in this heterogeneous disease. Future work looking specifically at the fibroblastic foci may help clarify this.

We observed differential expression of VEGF receptors and co-receptors in response to hypoxia. VEGFR1 protein expression was significantly upregulated in response to hypoxia in NF. A similar response to hypoxia was observed for FF but this was not statistically significant and may be explained by substantial variability observed in the response of individual FF populations. Hypoxic upregulation of VEGFR1 is a consistent finding in studies of various cells, which may relate to a hypoxia-inducible enhancer element within the VEGFR1 gene promoter region [17]. Whilst this increased VEGFR1 expression may simply reflect a negative feedback loop for VEGF-A signaling, a role for VEGFR1 in macrophage migration and activation in fibrosis has been proposed [18].

NP1 NF expression was downregulated in response to hypoxia. Existing studies report contradictory findings of both NP1 upregulation and downregulation in response to hypoxia depending on the cell type studied [19, 20]. Specific alveolar epithelial cell (AT) NP1 deletion has been shown to augment the apoptosis of ATI and ATII cells after exposure to oxidative stressors and have a role in maintenance of normal alveolar structure [21]. Current paradigms suggest that alveolar epithelial injury is the initiating factor in IPF [22]. It is possible therefore that the NP1 down-regulation in response to hypoxia may influence cell survival and contribute to the fibrogenic process.

Prolonged breathing of high concentrations of oxygen is associated with the development of acute lung injury [13]. The finding that FF panVEGF-A, VEGF-A_xxx_a and fibronectin mRNA are all increased in response to hyperoxia is interesting and suggests further work is required to investigate the possible pro-fibrotic effects of hyperoxia in IPF and the role of NP1 as a regulator of this process. This has important clinical relevance in IPF, as high flow oxygen is often used therapeutically during acute exacerbations.

We accept the study is of relatively small numbers of individual fibroblast populations but is comparable to several other studies in this field and reflects difficulties in obtaining tissue samples from these patients. The authors also acknowledge that HIF-1α may also be secreted by the alveolar epithelium. In vitro co-cultures of IPF-derived alveolar epithelium and fibroblasts would be desirable to further study the interaction of these cells in response to hypoxia and hyperoxia but there are several recognized practical limitations to this [23].

## Conclusion

This data reinforces our hypothesis that co-ordinated expression of VEGF-A isoforms/receptors are important in the development of pulmonary fibrosis, with support for hypoxia, hyperoxia and VEGF-A_xxx_a isoforms as drivers of fibrogenesis.

## Additional files

Additional file 1: Methodology and statistical analyses. (DOCX 23 kb)

Additional file 2: Primer sequences used for quantitative reverse transcriptase polymerase chain reaction (qRT-PCR). VEGFR1: Vascular endothelial growth factor receptor 1, VEGFR2: Vascular endothelial growth factor receptor 2, Neuropilin 1 and 2: NP1 and NP2, For: Forward, REV: Reverse. (JPEG 90 kb)

Additional file 3: Figure S2. a) Expression of HIF-1α in normal (NF) and fibrotic (FF) fibroblast cultures following exposure to hypoxic-like growth conditions with Cobalt Chloride. Representative western blot of NF and FF cultures treated with (HO) or without (N) Cobalt Chloride (CoCl_2_) for 24 h (above) with densitometric analysis (below). A specific band was detected for HIF-1α in cells exposed to CoCl_2_, that was absent in normoxic fibroblast cultures (*p < 0.05). Hypoxic-like growth conditions increased HIF-1α expression to a greater extent in NF compared to FF (*p < 0.05), unpaired t-test, *n* = 4 performed, *n* = 1 shown. Tubulin was used as the loading control. L: Protein Ladder, N: Normoxia, HO: Hypoxia. b) Quantitative RT-PCR of Fibronectin and Procollagen-1α mRNA in NF and FF total RNA lysates following exposure to hypoxia and hyperoxia. Fibronectin mRNA levels were significantly increased in total RNA lysates of NF and FF fibroblasts exposed to 24 h of hypoxia (NF *p < 0.05, FF **p < 0.01) and in FF exposed to 24 h of hyperoxia (*p < 0.05) when compared to normoxia using qRT-PCR. In contrast, hypoxia and hyperoxia had no significant effect on procollagen-1α mRNA levels. Data are presented as mean fold change in expression (2^-△△CT^) with SEM, data analysis performed on △△CT values (NF and FF *n* = 6). Statistical analysis: analysis of variance with post hoc Holm-Sidak multiple comparisons analysis used throughout. (JPEG 53 kb)

Additional file 4: Figure S3. Expression of VEGF-A receptor and co-receptor mRNA and proteins in response to hypoxia and hyperoxia in normal (NF) and fibrotic (FF) fibroblasts. a) Quantitative RT-PCR of VEGFR1, neuropilin (NP) 1, and NP2 mRNA expression in total RNA cell lysates in NF and FF. VEGFR1 (***p < 0.001) and NP2 (*p < 0.05) mRNA levels were significantly up-regulated in NF in response to exposure to hypoxia, whilst NF NP1 mRNA levels were significantly downregulated (*p < 0.05). Similarly, FF VEGFR1 (*p < 0.05) and NP2 (****p < 0.0001) mRNA levels were significantly upregulated in response to hypoxia, but NP1 mRNA levels were unaffected. Hyperoxia had no significant effect on VEGFR1 or NP2 mRNA levels in neither NF or FF, whilst hyperoxia significantly upregulated (*p < 0.05) FF NP1 mRNA levels. Data are presented as mean fold change in expression (2^-△△CT^) with SEM, data analysis performed on △△CT values (NF and FF *n* = 6). b) The effect of hypoxia and hyperoxia on VEGFR1 protein expression in NF and FF as measured by western blotting (above) and densitometric analysis (below). VEGFR1 protein expression was significantly upregulated (***p < 0.001) in response to hypoxia in NF but not in FF. Hyperoxia had no statistically significant effect on VEGFR1 expression in NF or FF. c) Hypoxia resulted in the significant down-regulation of NP1 protein expression in NF cell lysates (*p < 0.05), but had no significant effect on FF. Hyperoxia up-regulated NP1 protein expression in FF (p* < 0.05), but had no significant effect on NF. d) Hypoxia and hyperoxia had no significant effect in the expression of NP2 protein. Data presented as means with SEM (*n* = 4, *n* = 2 shown in each western blot image). Normal: Normal fibroblasts, Fibrotic: Fibrotic fibroblasts, N: Normoxia, HO: Hypoxia, HE: Hyperoxia. Tubulin: loading control. Analysis of variance with post hoc Dunnett’s multiple comparisons analysis used throughout. (JPEG 114 kb)
