# Supplementary material for: Effects of hypoxia and hyperoxia on the differential expression of VEGF-A isoforms and receptors in Idiopathic Pulmonary Fibrosis (IPF)
Source: Respir Res. 2018 Jan 15;19:9. doi: 10.1186/s12931-017-0711-x (PMC5769544; doi:10.1186/s12931-017-0711-x)
Supplement: Supplementary file 1 — Methodology and statistical analyses. (DOCX 23 kb) [file 12931_2017_711_MOESM1_ESM.docx]

**ON-LINE Supplement**

**Methodology**

**Lung Specimens**

Anonymised human lung biopsies were obtained from patients undergoing open or VATS thoracoscopy as part of their usual care. Tissue surplus to diagnostic requirements with either normal histological appearance or a pattern of UIP with clinical diagnosis of IPF were accepted. The institutional review boards of both North Bristol (NBT) and United Bristol Healthcare Trusts (UBHT) granted ethical approval for the study. Written informed consent was obtained from each patient.

**Cell culture**

*Primary Human Lung Fibroblasts*

Primary human lung fibroblasts (NF and FF n=4) were explanted from human lung specimens using established techniques. Briefly, the tissue was mechanically dissociated into fragments in sterile plastic dishes. Cells proliferated from the fragments under standard growth conditions (humidified atmosphere at 37^o^C in 95% air and 5% CO_2_) in DMEM-F12 media (Gibco Life Technologies) supplemented with 1% Fungizone (Gibco), 1% Peniciliin/Stretopmycin (Sigma) and 10% Heat inactivated Fetal Bovine serum (Gibco) for 4-6 weeks until cell confluency was achieved. Additional anonymised IPF fibroblast cultures were kindly donated by Dr Toby M. Maher (Royal Brompton Hospital, London, UK), NF and FF n=2. Normal and IPF fibroblast cultures (American Type Culture Collection, ATCC) were also purchased, NF and FF n=1. Fibroblasts were used for experiments between passages 3-9.

*Exposure of cells to Hypoxic and Hyperoxic growth conditions*

Cells were seeded into T25 flasks and grown to 70% confluency in standard growth conditions. They were serum starved for 24 hours, prior to incubation in one of three parallel experimental conditions: Standard growth conditions- Normoxia (N), Hypoxic-like (HO, 21%O_2_ with 0.5mM Cobalt chloride (CoCl2, Sigma) or Hyperoxia (HE, 90% O_2_ via a ProOx chamber BioSpherix, Model C21). After 24 hours incubation, total RNA was extracted or cell lysates prepared for subsequent analysis.

**RNA extraction**

Total cellular RNA was extracted from fibroblast lysates using the Quick RNA^TM^ mini prep kit (Zymo Research), following manufacturers’ recommendations to avoid gDNA contamination. RNA was eluted directly into RNase-free tubes and stored at -80^o^C until required. The Implen Nanophotometer^TM^ Pearl version 1.0 (Geneflow, Staffordshire, UK) was used to quantify the RNA product and ensure its purity.

**Quantitative RT-PCR**

Quantitative RT-PCR was used to determine mRNA levels of VEGF-A receptors, co-receptors and VEGF-A isoforms in response to varied oxygen exposure. First-strand cDNA synthesis was performed on 1µg RNA using the Taqman High Capacity RNA-to-cDNA kit (Applied Biosystems). Quantitative RT-PCR reaction mixes were made in accordance with manufacturer recommendations (SensiFAST SYBR Hi-ROX, Bioline).

Quantification of panVEGF-A, VEGF-A_xxx_a and VEGF-A_xxx_b mRNA were determined using previously published methodology^1^. Primer sequences used are detailed in S1.

The standard cycling regime consisted of 95^o^C for an initial 2 minutes, followed by cycles of 95^o^C for 5 seconds then 60^o^C for 30 seconds, for 50 cycles for identification of VEGF-A isoforms and 40 cycles for all other products. Minus reverse transcriptase and non-template control reactions were additionally performed as negative controls. The expression level of each gene was normalised to β-actin expression. Fluorescence data from each sample were analysed with the 2^-∆∆Ct^ method, where ∆∆Ct = (Ct GI unknown sample - Ct Actin unknown sample) - (Ct GI calibrator/control sample – Ct Actin calibrator /control sample), where GI is the gene of interest. Statistical analyses were performed on ∆∆Ct values.

**Western blotting**

Cell lysates were prepared from cell cultures in RIPA (Radio-Immunoprecipitation) extraction buffer (Sigma) containing protease and phosphatase inhibitors (Sigma). The protein concentration of cellular lysates was quantified using the BCA (Bicinchoninic acid) protein assay kit (Thermo Scientific). Protein (30-45µg) was re-suspended in Laemelli sample buffer (Sigma) heated to 100^o^C for 5 min then resolved by SDS-PAGE in 8-12% acrylamide gel. The proteins were transferred to PolyVinylidine DiFluoride (PVDF, Thermo-scientific, 0.45µm) membrane. After blocking the membrane in 3% bovine serum albumin in TBST for 1 hour at room temperature (RT), membranes were incubated overnight at 4^o^C with primary antibodies: mouse monoclonal anti-Tubulin-α (Fisher), anti-HIF-1α (BD Biosciences), VEGFR1 (Abcam), VEGFR2 (Cell Signaling), NP1 (Abcam), NP2 (Santa Cruz).

Membranes were then incubated with a HRP-conjugated secondary antibody (donkey anti-rabbit or sheep anti-mouse, GE Healthcare) in blocking buffer for 1 hour prior to visualisation by means of luminol-enhanced chemiluminescence (SuperSignal, West Femto, 34096, Thermo Scientific) and imaged with a UVP ChemiDoc-It imaging system (UVP, California, USA) and Visionworks LS software. Equal protein loading per lane was confirmed using tubulin controls. Semi-quantification of protein expression was undertaken by densitometry using Image-J software.

**ELISA**

Human PanVEGF-A and VEGF-A_xxx_b DUOset ELISA kits (R&D systems, DY3045 and DY293B respectively) were purchased to analyse total VEGF-A and VEGF-A_165_b isoform expression. Briefly, capture antibody (PanVEGF-A 1.0 µg/ml in PBS, VEGF-A_165_b 4.0 µg/ml in PBS) was added to 96-well plates (Clear Microplate, R&D systems) (at 100µl/well) and incubated overnight at RT. After three washes with PBST (400µl per well, PBS with 0.05% Tween 20, pH 7.2-7.4), plates were blocked with 300µl/well of blocking buffer (1% BSA/PBS) and incubated for 2 hours at RT. Then, 100µl/well of standard or sample, prepared in 1% BSA/PBS, were added to duplicate wells for 2 hours. After three washes, 100µl/well of detection antibody (working concentration of 100ng/ml in 1% BSA/PBS for PanVEGF-A, and 250ng/ml in 1% BSA/PBS for VEGF-A_165_b) was added and incubated at RT for two hours. Wells were washed again with PBST, then 100µl/well of HRP-conjugated streptavidin diluted 1:200 in 1% BSA/PBS was added and incubated for 20 minutes at RT, protected from light. Finally, after three more washes with PBST, 100µl/well of substrate solution was added (R&D Systems). The reaction was then stopped by addition of 100µl/well of Stop Solution (2M H_2_SO_4_) (R&D systems). The optical density of each well was determined using a microplate reader (Labsystems Mulitskan Plus, with Genesis software) at 450nm. The limit of detection of the panVEGF-A and VEGF-A_165_b ELISAs were calculated as 10pg/ml and 5-10pg/ml, respectively.

Total VEGFR2 expression was quantified in cell lysates using a DuoSet ELISA (R&D systems, DYC 1780) using the manufacturer’s protocol as outlined above. Specifics are highlighted. Capture antibody: 4.0 µg/ml in PBS, Wash buffer: PBS with 0.05%Tween 20, Blocking Buffer: 1%BSA, 0.05% NaN_3_ in PBS, pH 7.2-7.4), Reagent Diluent: 20mM Tris, 137mM NaCl, 0.05% Tween 20, 0.1% BSA, pH 7.2-7.4), Detection antibody: 200ng/ml in reagent diluent). The limit of detection of the ELISA was calculated to be 30pg/ml.

**Statistical analysis**

Statistical analysis was performed using GraphPad Prism version 5.0 software. The unpaired Student’s *t*-test was used to compare two groups, whilst the ANOVA with post hoc Holm’s Sidak multiple comparisons analysis was used for comparisons of multiple groups. A P value <0.05 was considered statistically significant.

**References**

1. Varey, AH, Rennel ES, Qiu Y *et al.* VEGF_165_b, an antiangiogenic VEGF-A isoform, binds and inhibits bevacizumab treatment in experimental colorectal carcinoma: balance of pro- and antiangiogenic VEGF-A isoforms has implications for therapy. *Br J Cancer 2008;* 98:1366-1379.
