# Supplementary figures and images for: Effects of hypoxia and hyperoxia on the differential expression of VEGF-A isoforms and receptors in Idiopathic Pulmonary Fibrosis (IPF)
Source: Respir Res. 2018 Jan 15;19:9. doi: 10.1186/s12931-017-0711-x (PMC5769544; doi:10.1186/s12931-017-0711-x)

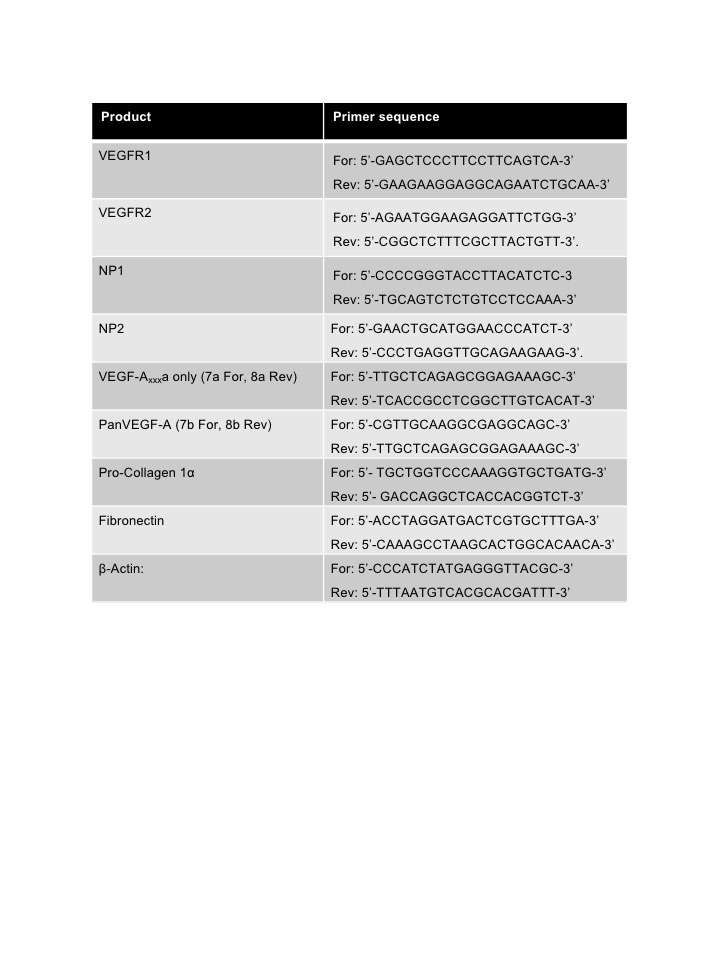

Supplement: Supplementary file 2 — Primer sequences used for quantitative reverse transcriptase polymerase chain reaction (qRT-PCR). VEGFR1: Vascular endothelial growth factor receptor 1, VEGFR2: Vascular endothelial growth factor receptor 2, Neuropilin 1 and 2: NP1 and NP2, For: Forward, REV: Reverse. (JPEG 90 kb) [file 12931_2017_711_MOESM2_ESM.jpg]

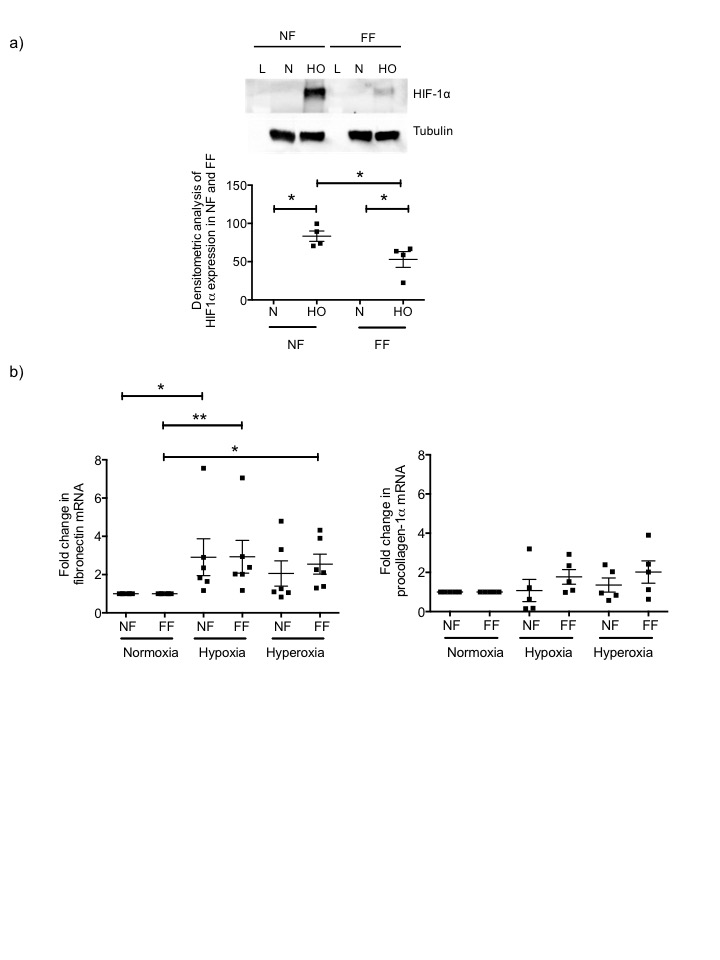

Supplement: Supplementary file 3 — a) Expression of HIF-1α in normal (NF) and fibrotic (FF) fibroblast cultures following exposure to hypoxic-like growth conditions with Cobalt Chloride. Representative western blot of NF and FF cultures treated with (HO) or without (N) Cobalt Chloride (CoCl2) for 24 h (above) with densitometric analysis (below). A specific band was detected for HIF-1α in cells exposed to CoCl2, that was absent in normoxic fibroblast cultures (*p < 0.05). Hypoxic-like growth conditions increased HIF-1α expression to a greater extent in NF compared to FF (*p < 0.05), unpaired t-test, n = 4 performed, n = 1 shown. Tubulin was used as the loading control. L: Protein Ladder, N: Normoxia, HO: Hypoxia. b) Quantitative RT-PCR of Fibronectin and Procollagen-1α mRNA in NF and FF total RNA lysates following exposure to hypoxia and hyperoxia. Fibronectin mRNA levels were significantly increased in total RNA lysates of NF and FF fibroblasts exposed to 24 h of hypoxia (NF *p < 0.05, FF **p < 0.01) and in FF exposed to 24 h of hyperoxia (*p < 0.05) when compared to normoxia using qRT-PCR. In contrast, hypoxia and hyperoxia had no significant effect on procollagen-1α mRNA levels. Data are presented as mean fold change in expression (2-△△CT) with SEM, data analysis performed on △△CT values (NF and FF n = 6). Statistical analysis: analysis of variance with post hoc Holm-Sidak multiple comparisons analysis used throughout. (JPEG 53 kb) [file 12931_2017_711_MOESM3_ESM.jpg]

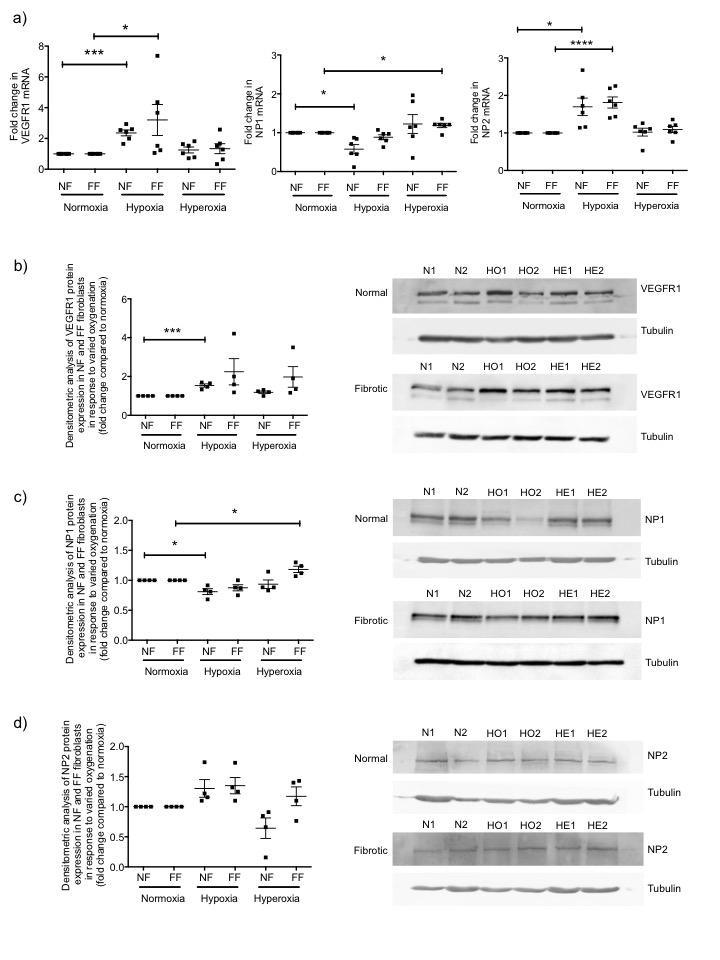

Supplement: Supplementary file 4 — Expression of VEGF-A receptor and co-receptor mRNA and proteins in response to hypoxia and hyperoxia in normal (NF) and fibrotic (FF) fibroblasts. a) Quantitative RT-PCR of VEGFR1, neuropilin (NP) 1, and NP2 mRNA expression in total RNA cell lysates in NF and FF. VEGFR1 (***p < 0.001) and NP2 (*p < 0.05) mRNA levels were significantly up-regulated in NF in response to exposure to hypoxia, whilst NF NP1 mRNA levels were significantly downregulated (*p < 0.05). Similarly, FF VEGFR1 (*p < 0.05) and NP2 (****p < 0.0001) mRNA levels were significantly upregulated in response to hypoxia, but NP1 mRNA levels were unaffected. Hyperoxia had no significant effect on VEGFR1 or NP2 mRNA levels in neither NF or FF, whilst hyperoxia significantly upregulated (*p < 0.05) FF NP1 mRNA levels. Data are presented as mean fold change in expression (2-△△CT) with SEM, data analysis performed on △△CT values (NF and FF n = 6). b) The effect of hypoxia and hyperoxia on VEGFR1 protein expression in NF and FF as measured by western blotting (above) and densitometric analysis (below). VEGFR1 protein expression was significantly upregulated (***p < 0.001) in response to hypoxia in NF but not in FF. Hyperoxia had no statistically significant effect on VEGFR1 expression in NF or FF. c) Hypoxia resulted in the significant down-regulation of NP1 protein expression in NF cell lysates (*p < 0.05), but had no significant effect on FF. Hyperoxia up-regulated NP1 protein expression in FF (p* < 0.05), but had no significant effect on NF. d) Hypoxia and hyperoxia had no significant effect in the expression of NP2 protein. Data presented as means with SEM (n = 4, n = 2 shown in each western blot image). Normal: Normal fibroblasts, Fibrotic: Fibrotic fibroblasts, N: Normoxia, HO: Hypoxia, HE: Hyperoxia. Tubulin: loading control. Analysis of variance with post hoc Dunnett’s multiple comparisons analysis used throughout. (JPEG 114 kb) [file 12931_2017_711_MOESM4_ESM.jpg]
